# A silent aggressor: disseminated histoplasmosis with laryngeal and adrenal involvement in a diabetic patient

**DOI:** 10.1530/EDM-25-0137

**Published:** 2026-04-02

**Authors:** Ayushi Singhal, Subhash B Yadav, Dharmendra Singh Bhadauria, Rungmei S K Marak, Manoj Jain

**Affiliations:** ^1^ Department of Endocrinology, Sanjay Gandhi Postgraduate Institute of Medical Sciences, Lucknow, India; ^2^ Department of Nephrology, Sanjay Gandhi Postgraduate Institute of Medical Sciences, Lucknow, India; ^3^ Department of Microbiology, Sanjay Gandhi Postgraduate Institute of Medical Sciences, Lucknow, India; ^4^ Department of Pathology, Sanjay Gandhi Postgraduate Institute of Medical Sciences, Lucknow, India

**Keywords:** diabetes, adrenal, *Histoplasma capsulatum*, granulomatous disease, vocal cord

## Abstract

**Summary:**

A 65-year-old male with poorly controlled type 2 diabetes mellitus presented with 7 months of progressive hoarseness of voice, fever, anorexia, cough, and weight loss. Just before admission, he developed drowsiness and memory impairment. Examination showed a drowsy but stable patient without meningeal signs and hyperpigmentation. Persistent hoarseness prompted laryngoscopy, which revealed a vocal cord nodule. Imaging demonstrated bilateral adrenal enlargement with hypodense lesions in liver, pancreas, spleen, and kidney. Hormonal evaluation showed preserved adrenal function. Histopathological examination of vocal cord tissue and an adrenal biopsy confirmed the diagnosis of disseminated histoplasmosis. The patient was treated with antifungal therapy. Over 18 months of therapy, adrenal function remained normal, renal function stabilized, and urinary histoplasma antigen became undetectable. Glycemic control improved on oral hypoglycemic agents, vocal recovery occurred with therapy, and the patient achieved long-term clinical stability.

**Learning points:**

- Disseminated histoplasmosis can present with hoarseness, fever, weight loss and multi-organ involvement.
- Adrenal enlargement is common but may not always cause insufficiency.
- Histopathology and fungal stains (PAS, GMS, and LPCB) are diagnostic gold standards.
- High clinical suspicion is needed in endemic regions or immunocompromised hosts.

## Background

Histoplasmosis is caused by *Histoplasma capsulatum*, a dimorphic fungus endemic in temperate regions, including India’s western and Gangetic regions (1). Infection occurs through inhalation of fungal spores from bird/bat droppings. While immunocompetent individuals often remain asymptomatic, immunocompromised patients, particularly those on immunosuppressants or with diabetes, are at risk of disseminated disease. Chronic hyperglycemia impairs neutrophil function, cytokine response, and cell-mediated immunity (2). In severe cases, histoplasmosis can spread to other organs, such as the liver, spleen, bone marrow, skin, adrenal glands, and kidneys. Vocal cord involvement is rare, and its diagnosis requires a high index of suspicion (3).

Here, we report a 65-year-old diabetic patient with hoarseness, fever, and altered sensorium, later diagnosed with disseminated histoplasmosis involving bilateral adrenals and vocal cords, which was resolved with antifungal medications (amphotericin B and itraconazole).

## Case presentation

A 65-year-old patient with poorly controlled type 2 diabetes (glycated hemoglobin: 9.2% (77 mmol/mol)) presented with a 7-month history of progressive hoarseness of voice and intermittent high-grade fever and a 3-month history of anorexia and dry cough, with a significant weight loss (15 kg). Three days before admission, the patient developed drowsiness and memory impairment. Examination revealed a drowsy but hemodynamically stable patient without meningeal signs. There were no features of hyperpigmentation.

## Investigation

Patient’s hematological and biochemical investigations were done (Table 1).

**Table 1:** Hemogram and biochemical investigations at presentation and during follow-up.

Parameters	Baseline	Seven days after AFT	Reference range
Hemoglobin, g/L	104	100	120–160
Total leukocyte count/mm^3^	3,100	5,500	4,000–10,000
Platelet count × 10^5^/mm^3^	1.1	3.3	1.5–4.5
Serum creatinine, μmol/L	150.3	114.9	44.2–141.4
Serum sodium, mmol/L	129	138	135–145
Serum potassium, mmol/L	3.1	3.5	3.5–4.5
Corrected serum calcium, mmol/L	3.2	2.7	2.12–2.69
Serum magnesium, mmol/L	0.7	0.74	0.74–1.07
Serum phosphorus, mmol/L	1.58	1.1	0.81–1.45
Alkaline phosphate, U/L	210	160	35–150
24 h urinary protein, g/day	1.2	ND	<0.15
Serum procalcitonin, μg/L	0.59	ND	<0.5
CD4 count/mm^3^	302	ND	>250
Beta-2 microglobulin, nmol/L	466.5	ND	59.-100.08
Kappa/lambda chain	1.05	ND	0.26–1.65
Serum electrophoresis	Faint M-band in gamma region		
Urine culture	+ve		

CD4, cluster of differentiation 4; SGPT, serum glutamic pyruvic transaminase; SGOT, serum glutamic oxaloacetic transaminase; AFT, antifungal treatment; +ve, urine culture shows growth of histoplasma after >4 weeks of incubation.

**Table 2:** Hormonal investigations at presentation and during follow-up.

Parameters	Baseline	Seven days after AFT	At discharge	Six months after discharge	Normal range
Serum cortisol, nmol/L	509	280	337	513	166.0–507.0
ACTH, pmol/L	11	ND	ND	ND	1.6–13.9
Plasma renin activity, ng/mL/h	0.3	ND	ND	0.9	0.3–1.9
HbA1c, mmol/mol	77	ND	ND	66	20–38
iPTH, pmol/L	0.65	ND	ND	ND	1.6–6.9
25-Hydroxyvitamin D, nmol/L	57	ND	ND	ND	50–250

ACTH, adrenocorticotrophic hormone; HbA1c, glycated hemoglobin; iPTH, intact parathyroid hormone; ND, not done; AFT, antifungal treatment.

Initial workup showed bicytopenia, a faint M-band and elevated beta-2 microglobulin. However, serum and urinary immunofixation studies did not indicate monoclonal gammopathy. Bone marrow aspiration and biopsy ruled out plasma cell disorder.

**Figure 1:**
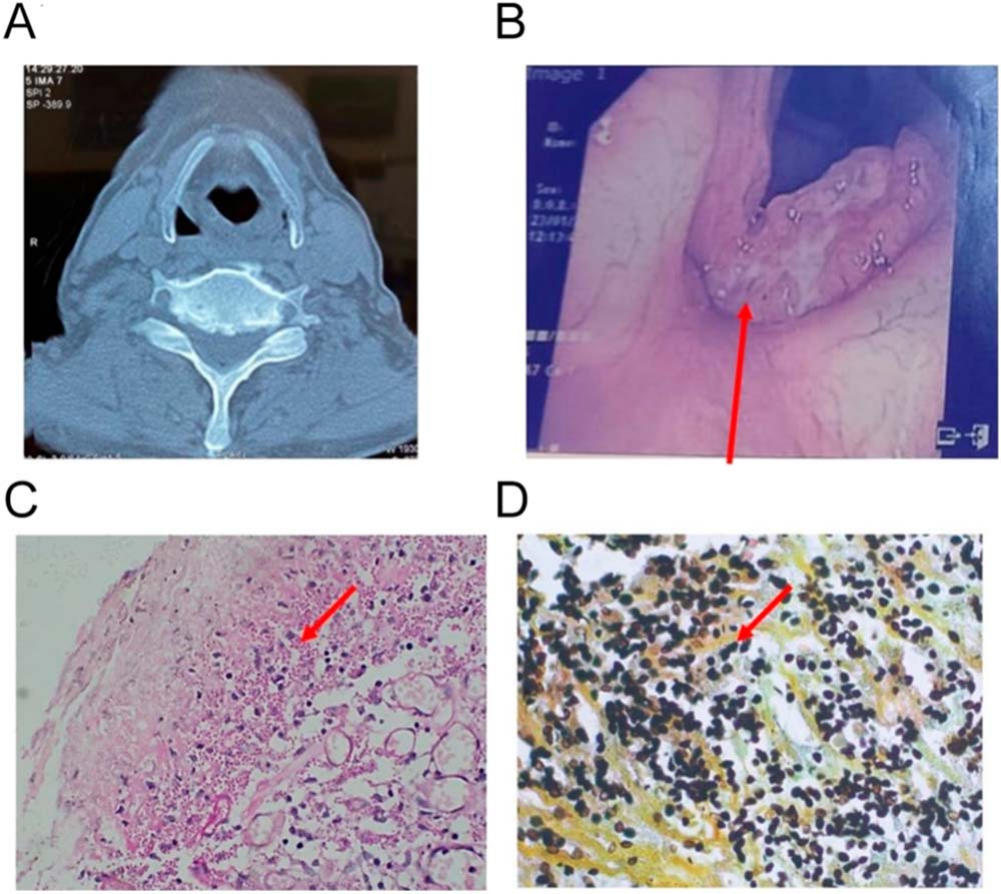
(A) Contrast-enhanced computed tomography (CECT) neck image showing bulky bilateral vocal cord (left > right) with obliteration of pyriform fossa on left side. (B) Laryngoscopy image showing growth of left vocal cord, eroding the anterior commissure. (C) Small 2–5 microns budding, intracellular yeast (arrow) in vocal cord tissue, the periodic acid–Schiff (PAS) stain, 100× original magnification. (D) Small 2–5 microns budding, intracellular yeast (arrow) in vocal cord tissue. Grocott–Gömöri’s methenamine silver (GMS) stain, 1,000× original magnification.

Persistent hoarseness led to a neck contrast-enhanced computed tomography (CECT) and a laryngoscopy identified a vocal cord nodule (Fig. 1A and B). Histopathology of the excised lesion revealed small, intracellular budding yeast (2–5 microns) on periodic acid–Schiff (PAS) stain and Grocott–Gömöri’s methenamine silver (GMS) stain, confirming *Histoplasma capsulatum* infection (Fig. 1C and D). The patient reported pigeons exposure but no recent travel history.

**Figure 2:**
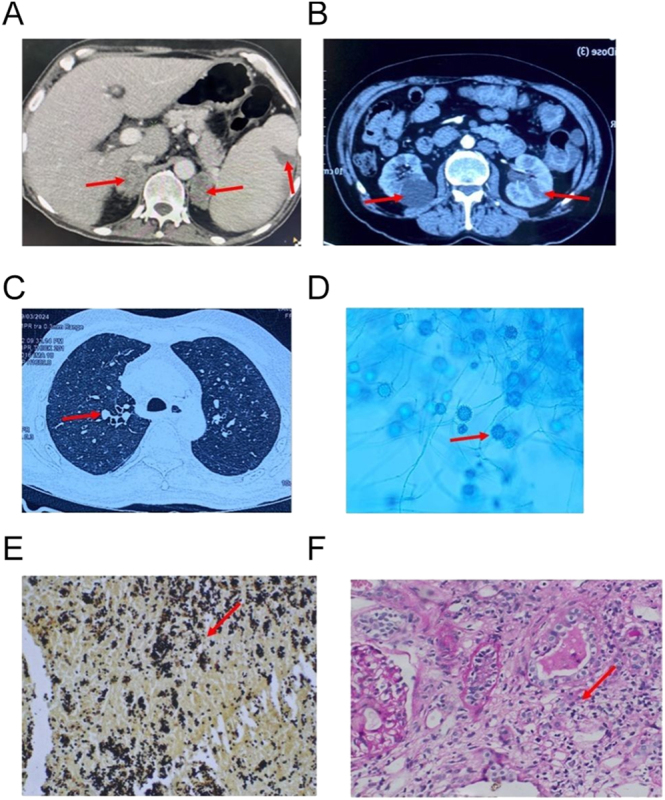
(A) CECT abdomen image showing bilateral enlarged adrenal with central hypodensity due to necrosis and/or hemorrhage along with bulky spleen with infarct. (B) The CECT abdomen image shows multiple small hypodense lesions in the pancreas and kidneys. (C) High-resolution computed tomography (HRCT) showing bilateral lung nodules. (D) Lactophenol cotton blue (LPCB) mount shows thin hyaline septate branching fungal hyphae and plenty of tuberculate macroconidia and a few microconidia, 40× original magnification. (E) Small 2–5 microns budding, intracellular yeast (arrow) in adrenal cord tissue. GMS stain, 400× original magnification. (F) Kidney biopsy showing moderate mixed tubule interstitial infiltrate suggestive of tubulointerstitial nephritis. PAS stain, 400× original magnification.

Further imaging (CECT of the abdomen and high-resolution CT of the lungs) demonstrated bilateral adrenal enlargement (4.5 cm) with hypodense foci, and hypodense lesions in the liver, pancreas, spleen, and kidney, suggesting disseminated histoplasmosis (Fig. 2A, B, C). Despite adrenal involvement, there was no evidence of mineralocorticoid or glucocorticoid deficiency (08:00 h serum cortisol: 509 nmol/L, normal range (NR): 166–507 nmol/L; plasma adrenocorticotropin: 11.0 pmol/L, NR: 1.6–13.9 pmol/L; and plasma renin activity of 0.3 ng/mL/h (NR: 0.3–1.9 ng/mL/h)) (Table 2).

A CT-guided adrenal biopsy confirmed *H. capsulatum* with 10% potassium hydroxide (KOH) mount and lactophenol cotton blue (LPCB) staining, showing characteristics of tuberculate macroconidia (Fig. 2D). Histopathology of adrenal tissue confirmed intracellular yeast of *Histoplasma capsulatum* using hematoxylin and eosin (H&E) stain and GMS stain (Fig. 2E). A GeneXpert assay (a cartridge-based nucleic acid amplification test) and culture for tuberculosis on adrenal tissue were negative.

To evaluate for an immunocompromised state predisposing to disseminated histoplasmosis, relevant tests were performed. Viral serologies (hepatitis B, hepatitis C, and human immunodeficiency virus) were non-reactive;. CD4 counts were within the normal range.

Persistent proteinuria (24 h urinary protein: 1.2 g/day; normal <0.15 g/day) prompted a renal biopsy, revealing tubulointerstitial nephritis likely histoplasma induced (Fig. 2F). Immunohistochemistry (IHC) was performed on kidney biopsy tissue to evaluate the etiology of interstitial nephritis and was negative for IgG, IgM, IgA, C3, C1q, kappa light chains, and lambda light chains. However, urine fungal culture showed growth of histoplasma after 4 weeks of incubationsuggesting direct renal invasion by the fungus. Due to altered sensorium, magnetic resonance imaging (MRI) of brain and cerebrospinal fluid (CSF) analysis were performed, showing nonspecific pontine hypointensities on T2/FLAIR but no CSF abnormalities.

## Treatment

The patient received intravenous liposomal amphotericin B (3 mg/kg) for 4 weeks followed by oral itraconazole (200 mg three times a day for 3 days and then 200 mg twice a day) for 18 months. Glycemic control was managed with a basal bolus insulin regimen and later switched to oral agents due to recurrent hypoglycemia. Additionally, the patient was found to have parathyroid hormone (PTH) independent hypercalcemia, with a corrected calcium of 3.2 mmol/L (NR: 2.1–2.7 mmol/L) and a low serum PTH of 0.65 pmol/L (NR: 1.6–6.9 pmol/L). This was managed with intravenous fluids and zoledronic acid.

## Outcome and follow-up

After 18 months of itraconazole adrenal function remained normal and urinary Histoplasma antigen became undetectable (Table 2). Fundus examination showed no features of diabetic retinopathy. Follow-up serum creatinine was 114.9 μmol/L (reference range: 44.2–141.4 μmol/L). Insulin therapy was discontinued owing to recurrent hypoglycemia despite minimal insulin requirements, and glycemic control was successfully transitioned to oral hypoglycemic agents. Speech therapy improved vocal recovery, and patient remains clinically stable with optimized metabolic management.

## Discussion

Disseminated histoplasmosis, a life-threatening fungal infection spreads beyond the lungs. It primarily affects immunocompromised individuals, while immunocompetent patients often have self-limited or asymptomatic course. Symptoms may include malaise, fever, and dyspnea. Disease severity depends on immune status and fungal exposure (2, 4). Diabetic- patients face a higher risk due to defective cellular immunity (2, 5).

Disseminated histoplasmosis commonly affects liver, spleen, and bone marrow, with additional involvement of the lungs, skin, adrenal glands, central nervous system, and gastrointestinal tract (1). Laryngeal histoplasmosis is rare and typically occurs in disseminated disease, often mimicking laryngeal carcinoma or tuberculosis (3). Symptoms include hoarseness, dysphagia, odynophagia, sore throat, dyspnea, lethargy, and weight loss. Direct laryngoscopy reveals pearl-white granulomas, ulcerative or verrucous lesions, commonly on the false cords, and aryepiglottic folds. Diagnosis requires a biopsy or swab of the lesion for culture and histopathological examination aided by special stains, such as H&E, GMS, and periodic acid–Schiff (PAS), for identifying *Histoplasma* organisms (3).

Our patient had persistent proteinuria (1.2 g per day). Kidney involvement often presented as granulomatous interstitial nephritis, detectable by GMS stain, while chronic disease may progress to non-granulomatous tubulointerstitial nephritis. Recognizing these patterns is essential for accurate diagnosis and appropriate management of renal involvement in histoplasmosis (6). A positive urine culture for *Histoplasma capsulatum* in a patient with disseminated histoplasmosis and non-granulomatous interstitial nephritis is highly significant, as it provides definite culture-based evidence of direct renal invasion by the fungus (6).

Hypercalcemia, although uncommon, results from granuloma-induced overproduction of 1,25(OH)_2_D due to unregulated 1-alpha-hydroxylase in macrophages (7). This mechanism more typical for sarcoidosis and tuberculosis, also occurs in fungal infections-, such as histoplasmosis, candidiasis, cryptococcosis, paracoccidioidomycosis and pneumocystosis (7).

Our patient had bilateral adrenal gland enlargement on CT, with *Histoplasma capsulatum* confirmed on biopsy, but had no adrenal insufficiency. This aligns with the previous studies reporting adrenal insufficiency in 10–30% of adrenal histoplasmosis cases. Linder & Kauffman noted that insufficiency is uncommon unless both glands are fully replaced by necrotizing granulomatous inflammation (8). Adrenal function does not recover with antifungal treatment; it requires lifelong corticosteroid supplementation (9). Adrenal insufficiency may also arise later in treatment due to disease-related adrenal atrophy and calcification, as well as the effects of antifungal therapy, especially ketoconazole which disrupts steroid synthesis. Hence, regular monitoring of cortisol and plasma renin activity during follow-up is essential (9).

The Infectious Diseases Society of America (IDSA) recommends liposomal amphotericin B (3.0 mg/kg/day for 1–2 weeks), followed by oral itraconazole (200 mg three times daily for 3 days and then 200 mg twice daily for at least 12 months) for the progressive disseminated histoplasmosis. Galgiani *et al.* suggested intravenous (IV) liposomal amphotericin B in cases of progressive disseminated histoplasmosis until clinical improvement is achieved (10). For central nervous system histoplasmosis, a higher-dose of liposomal amphotericin B (5.0 mg/kg/day for a total of 175 mg/kg over 4–6 weeks) was suggested, followed by itraconazole for at least one year until the resolution of CSF abnormalities and normalization of histoplasma antigen levels. Itraconazole levels (2–4 μg/mL) should be monitored by high-performance liquid chromatography (HPLC) to ensure efficacy and avoid toxicity (10, 11).

Although a confirmatory diagnosis of histoplasma requires histopathological examination or culture, other investigations, such as antigen detection, may aid in diagnosis. Additionally, testing for the Histoplasma galactomannan antigen in urine and serum is valuable for monitoring treatment response and detecting relapses, with urine detection being more sensitive than serum (12).

## Conclusion

Diabetic patients with signs and symptoms of systemic infection need opportunistic infection screening, particularly in endemic region. Laryngeal histoplasmosis is a rare entity mimicking carcinoma and tuberculosis. Histopathology or culture confirms diagnosis; antifungals are primary treatment. Monitoring adrenal function and itraconazole levels is mandatory for optimal care.

## Declaration of interest

The authors declare that there is no conflict of interest that could be perceived as prejudicing the impartiality of the research reported. 

## Funding

This research did not receive any specific grant from any funding agency in the public, commercial, or not-for-profit sector.

## Patient consent

Written informed consent for publication of their clinical details was obtained from the patient.

## Author contribution statement

All authors made individual contributions to the preparation of this manuscript. AS, SBY, and DSB were involved in the diagnosis and clinical management of the patient, as well as in drafting and submitting the manuscript. RSKM contributed to the microbiological investigations, including mycological confirmation. MJ was responsible for the histopathological analysis and preparation of histology images. All authors have reviewed and approved the final version of the manuscript.

## Patient’s perspective

I had persistent change in voice along with intermittent high-grade fever, which troubled me a lot. I consulted many local doctors and received treatment, but there was no relief. My anxiety increased when I noticed progressive weight loss and a marked reduction in appetite. Through a relative, I came to this referral care center. I was told that my CT scan had a nodule in the voice box and enlargement of both adrenal glands. A biopsy confirmed a fungal infection. I became very anxious as my health kept worsening. With help from my relative, the doctors explained my illness and how serious it was because it had spread throughout my body. They started me on a medicine called amphotericin for fungal infections. During the injections, I often got chills and shivering, but after about two weeks, I began to feel better, and my fever came down. I stayed in the hospital for five weeks, and by the time I was discharged, I had improved by almost 75%. After completing 18 months of antifungal treatment, my medicines have stopped. I feel energetic again, my speech is much better, and I am thankful to the doctors and staff for their great care during my stay in the endocrinology ward.
